# Diabetes mellitus and associated factors among psychiatric patients receiving antipsychotic treatment at Wachemo University Nigist Elleni Hospital, South Ethiopia

**DOI:** 10.1371/journal.pone.0333284

**Published:** 2025-10-07

**Authors:** Amanuel Melaku Laemango, Misgana Bekele Begna, Dembelo Tirago Masebo, Dilebo Lefebo Anshebo, Shifarew Bekele Woyesa, Mengistu Lodebo Funga

**Affiliations:** 1 Hosanna College of Health Sciences, Medical Laboratory department,; 2 Jimma University school of medical laboratory,; 3 Hosanna College of Health Sciences, Midwifery Department; University of Thessaly Faculty of Medicine: Panepistemio Thessalias Tmema Iatrikes, GREECE

## Abstract

**Background:**

Diabetes mellitus is one of the most common non-communicable diseases, and its epidemic proportion has placed it at the forefront of public health challenges currently facing the world. Patients with severe mental illnesses who receive antipsychotic treatment have a high risk of developing metabolic disturbances.

**Method and material:**

A hospital-based cross-sectional study was conducted. A total of 216 psychiatric patients on treatment were included and selected by consecutive convenient sampling techniques. An interviewer-administered questionnaire was used to collect socio-demographic, clinical, and behavioral characteristics. The levels of fasting blood glucose were measured by Cobas 4000 series Cobas c311Basel, Switzerland. The data were entered into Epi data version 4.6 and analyzed by SPSS version 25. Data were summarized by tables, graphs, and descriptive statistics. Bivariate and multivariate logistic regressions were used. P-value <0.05 was considered statistically significant.

**Result:**

A total of 216 psychiatric patients were enrolled in this study. The overall prevalence of diabetes mellitus was 25(11.6%). Among participants with diabetes mellitus, the prevalence of diabetes mellitus was higher in females than males (17(68.0%) vs 8(32.0%). Female (AOR:6.483, 95% CI (1.647, 25.516), P- value = 0.007), longer duration of antipsychotic use (AOR: 7.876 (1.587–39.090), moderate physical activity (AOR: 0.220, 95% CI (.057-.85), P- value = 0.028), higher body mass index (11.869, 95% CI (2.188–64.374), P- value = .004), and high cholesterol(AOR:13.742. 95% CI (3.153–59.887), P- value = .000) were significantly associated to diabetes mellitus.

**Conclusion:**

The finding of this study revealed that the prevalence of diabetes mellitus was 11.6%. Female sex, longer duration of antipsychotic use, moderate physical activity, overweight BMI, and high total cholesterol were significantly associated. Therefore, Health providers should regularly weigh patients and monitor long-term side effects of medication.

## Introduction

Diabetes mellitus (DM) is a heterogeneous group of metabolic disorders characterized by persistent hyperglycemia and disturbances in carbohydrate, fat, and protein metabolism secondary to abnormalities in insulin secretion, action, or both. Type 1 diabetes mellitus, type 2 diabetes mellitus (T2DM), gestational diabetes, and other types of diabetes are the four forms of the disease [1,2]. There are complex genetic, epigenetics, environmental, and behavioral factors that contribute to the development of diabetes [3].

A psychiatric disorder is a clinically significant behavioural or psychological condition or pattern that develops in an individual and is connected with current suffering or incapacity [4]. Over time and in all situations, patients with serious mental illness consistently have higher mortality rates than the general population [5–7].

When receiving antipsychotic medication for serious mental diseases, patients are at an increased risk of acquiring metabolic disorders such as obesity, type 2 diabetes, dyslipidemia, hypertension, and metabolic syndrome [8,9]. Associated factors for DM in people with mental disorders include increasing age, prolonged duration of illness, and a diagnosis of schizophrenia, the latter being an independent predictor of DM in people with mental disorders [10]. Environmental variables, including poverty and aspects of the mental illness, as well as poor lifestyle choices (diets high in refined sugar and saturated fat combined with little physical activity), contribute to the physical illness [11].

Antipsychotic drugs (APDs) are the cornerstone of treatment for schizophrenia and other mental diseases, including bipolar disorders, dementia, autism-related irritability, and severe mental illness [12,13]. Therapeutic effects of typical APDs are mediated largely through a potent block of dopamine D2 receptors, which also cause extra-pyramidal symptoms side effects [14]. Atypical Antipsychotics, or (SGAs, are new medications that were approved for use in the 1990s. clozapine, olanzapine, amisulpride quetiapine risperidone ziprasidone aripiprazole are atypical antipsychotic drugs [11,12,15]. SGAs are now widely used as first-line APDs due to their improved tolerability and reduced extrapyramidal symptoms compared with typical APDs [14]. The rates of metabolic syndrome, dyslipidemia, and diabetes mellitus are significantly higher in people taking atypical antipsychotics than in those taking conventional antipsychotics or mood stabilizers to treat schizophrenia or bipolar disorder [12,15–18].

Antipsychotics act on glucose and insulin homeostasis in a variety of direct and indirect mechanisms such as the effect of antipsychotic-induced weight gain on insulin resistance, a direct effect on insulin signaling, and a direct cytotoxic effect on the pancreatic β-cell [11,12,17]. APDs can act as an antagonists of serotonin 5-HT2C, histamine H1, and dopamine D2 receptors to increase appetite and thus increase food intake leading to obesity. It can also direct cytotoxic effect on pancreatic β-cell by the mitochondrial apoptotic pathway [19].

People with severe mental illness are more susceptible to the negative effects of type 2 diabetes mellitus (T2DM), which is highly predictive of cardiovascular diseases [20,21]. In people with schizophrenia, diabetes risk is correlated with heredity, antipsychotic medication, poor food habits, and lack of physical activity. A higher death rate, macrovascular (acute myocardial infarction, cerebrovascular accident, and peripheral vascular disease), microvascular (nephropathy, retinopathy, and neuropathy), and diabetic foot problems are linked to diabetes mellitus [22].

The study conducted in South Africa indicated prevalence 32% hypertension and 8% type-2 diabetes among long-term psychiatric patients, respectively [5]. The research carried out in Gondar and Hawassa revealed that the prevalence of diabetes among psychiatric patients was 7.3% and 6.3% respectively. Besides, diabetes was higher among patients receiving combined antipsychotic agents when compared to those receiving a single type of treatment agent [1,9s].

The prevalence of DM in psychiatric patients receiving antipsychotic treatment is increasing dramatically. Despite being conducted in specific areas, the prevalence of diabetes can vary due to a variety of factors such as patient behavior, lifestyle, genetic predisposition, environmental influences, and the clinical conditions of psychiatric patients. However, as far as our knowledge is concerned there is a paucity of research on DM in psychiatric patients receiving antipsychotic treatment, and no study was conducted in the study area. Therefore, this study aimed to fill this gap by assessing the prevalence of DM and its associated factors among psychiatric patients receiving antipsychotic treatment.

## Methods and materials

### Study setting and study population

**Fig 1:**
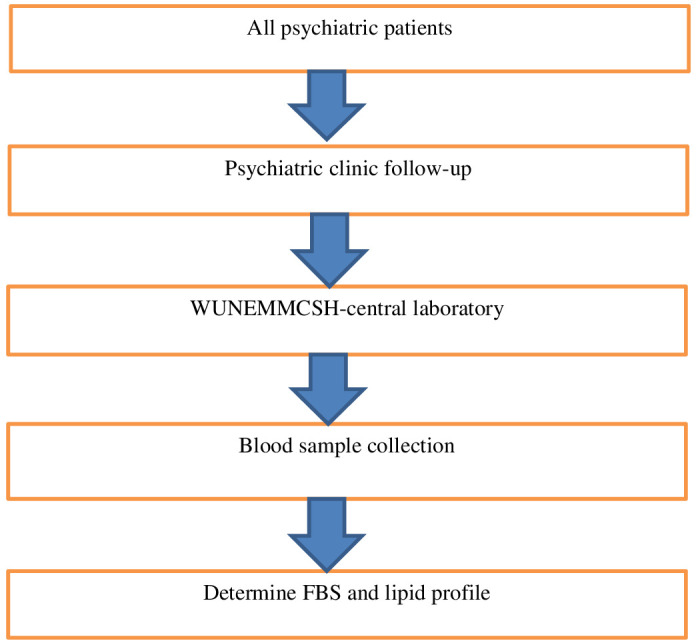
A flow chart of the participants included in the study.

The study was conducted at Wachemo University Nigist Eleni Mohammed Memorial Comprehensive Specialized Hospital (WUNEMMCSH) which is located in Hosanna town, Hadiya Zone, southern, Ethiopia. The town is 232 km far from the capital city Addis Ababa due north and 157 km from the regional city Hawassa due southeast. A hospital-based cross-sectional study was conducted to determine the prevalence of diabetes mellitus and associated factors among psychiatric patients receiving antipsychotic treatment. Adult psychiatric patients with SMI, those attending psychiatry care treatment service follow-up clinic and had two monthly regular follow-ups, at least for one year (12 months) follow up or on treatment and voluntarily giving informed consent were eligible for the study. Patients with previously diagnosed and known type 2 diabetes, HTN, pregnant women, patients on lipid-lowering drugs, known with chronic liver disease, and confirmed hypothyroidism were excluded (Fig 1).

### Sample Size and Sampling Technique

The sample size assumption was based on a 16% proportion of type-2 diabetes among psychiatric patients from a study conducted in the UK using a single population proportion formula at a 95% confidence interval (CI) and a marginal error of 5% [23].


$$n=\frac{({Z_{\alpha /2})}^{2}.\;pq}{({d)}^{2}}$$

Where, P = proportion of diabetes mellitus, Zα/2 = critical value at 95%, level of confidence (Z = 1.96), d = margin of error (5%), n = the required sample size, which is 207. However, the number of source populations is < 10,000, so a sample size correction was performed using Cochran’s formula for sample size correction. A final sample size of 216 was then calculated. Finally, a consecutive sampling technique was applied to select and include the study participants.

### Assessments and measurements

Socio-demographic data like age, sex, education, marital status, ethnicity, occupation, and religion; behavioral data such as khat chewing, smoking, and alcohol taking and clinical data like history of diabetes mellitus, type of mental disorder, duration of disease, and type of treatments were collected by the interviewer-administered structured questionnaires.

Before measuring BP, each patient was asked about alcohol/caffeine intake, smoking, stressful condition, and bathing for 30 min before taking a measurement. Measurements were taken from each patient by using a digital electronic sphygmomanometer.

Weight and height measurements were taken from the individuals based on the WHO step-wise guideline using an Adult's digital electronic scale that has both weight and height scales. Body mass index (BMI) was calculated as (weight/height2) and classified as BMI < 18.5 kg/m2 for underweight, 18.5–24.9 Kg/ m2 for normal weight, 25–29.9 kg/m2 for overweight and≥30 kg/m2 for obesity. Furthermore, the WHO step-wise technique was applied to measure the waist circumference (WC) of the patients with non-stretching tape [24]. Hip circumference (HC) was measured over light clothing at the widest portion of the buttocks as the participants are in a standing position and both feet together. Waist-to-hip ratio (WHR) was calculated by dividing WC by HC. While the cut-off point considered for WC is ≥ 80 cm for females and ≥94 cm for males to define overweight, the cut-off value taken for WHR will be > 0.8 for females and >0.9 for males as per the criterion of the WHO [24,25].

The study subjects were instructed about the procedure and the site for blood collection was selected preferably at the median cubital vein. A tourniquet was applied at 4–5 finger widths above the select vein puncture site. The sites were disinfected using 70% alcohol swabs for 30 seconds in a circular motion from inside to outside fashion and allowed to dry completely for 30 seconds. About 5 ml of venous blood was collected aseptically from the median cubital vein of each study participant by trained Laboratory Technologists in the morning after overnight fast. The collected blood sample was left for 30 minutes and centrifuged for 5 minutes at 3000 revolutions per minute (rpm) to separate serum from formed elements. The fasting blood glucose and lipid profiles (TC, HDL-C, LDL-C, TG) were analyzed Cobas 4000 series Cobas c311 (Basel, Switzerland) analyzer. FPG level, **≥ 126 mg/dl** in at least two occasions is a diagnostic for DM [26].

Standard quality control (normal and pathological) protocols were performed and passed before running the participants sample analysis to assure the accuracy and functionality of the instrument. The control result was be fallen within the acceptable ranges (mean±2SD). All necessary procedures and steps were followed based on the clinical chemistry SOP. The printed results were checked for a unit of reporting, the correctness of the medical registration number, and the unique identification card number.

### Statistical analysis

All the data were checked for completeness, cleaning, processing, and analysis of the data obtained from laboratory analysis of the blood sample. The data was coded and entered into Epi-data statistical software (version 3.1, 2008) and then it was exported to SPSS software (version 25.0, 2013, USA) for analysis. Descriptive statistics such as frequency and percentages were applied to summarize categorical variables. Besides normally distributed continuous variables were tabulated via means and standard deviation was applied for data with skewed distribution. Bivariate and multivariate logistic regression models also were analyzed to find out the independent factors that affect FBS and to determine significant predictors. It was accepted as statistically significant when a p-value <0.05 at 95% CI. Multicollinearity was checked by Spearman correlation and VIF.

### Data quality assurance and management

The data collection started after the questionnaire was Pre-tested on 5% of individuals in the psychiatry department to see the validity and completeness. Then correction was taken and these participants were excluded from the study. Training for data collectors was given on the study objective, procedures, confidentiality, respondent rights, and informed consent by the investigator, and the consistency of the data was checked. The filled questionnaires were checked daily for completeness, logical errors, unclear information, or irrelevant information

### Ethical consideration

Ethical clearance was obtained from the Institutional Review Board of Health Sciences faculty, at Jimma University. A letter of cooperation was written from the Jimma University research coordination office to WUNEMMCSH. The purpose, benefit, and method of the study were clearly explained to a participant in a language they can understand. All participants were informed that their responses would be kept confidential.

The written informed consent was taken from the participants and those who have the willingness to participate in the study were included. Participation in the study was voluntary and refusal was permitted. To ensure the confidentiality of data, study participants were identified using codes and unauthorized persons were not able to access the collected data. In addition, the clinical specimen collected during the study period was used for the stated objectives only. The study participant results were reported to the physician for proper management as necessary.

## Result

### Socio-demographic characteristics of the study participants

The study comprised 216 psychiatric patients who were on antipsychotic medications. One hundred thirteen (52.3%) of the total study participants, were men. The average age of the study participants was 34.15 ± (10.820), and 114 (52.8%) of them were from rural areas. Furthermore, 94 (43.5%) of the participants identified as protestants, 50 (23.1%) as jobless, 99 (45.8%) as married, 131 (60.6%) as Hadiya in ethnicity, 195 (90.3%) as literate, and 82 (42.3%) as having completed secondary education (**Table 1).**

**Table 1:** Socio-demographic characteristics of psychiatric patients receiving antipsychotic treatment at the Wachamo University Nigist Elleni Mohammed Memorial Comprehensive Specialized Hospital, Hossana, SNNPR, Ethiopia, June 01 to August 30, 2022.

Variable	Category	Diabetes mellitus		Total No (%)
		Yes No (%)	No No (%)	
AGE	≤20	1(4.0)	14(7.3)	15(6.9)
	21-30	5(20.0)	66(34.6)	71(32.9)
	31-40	5(20.0)	70(36.6)	75(34.7)
	41-50	8(32.0)	31(16.2)	39(18.1)
	≥50	6(24.0)	10(5.2)	16(7.4)
Sex	Male	8(32.0)	105(55.0)	113(52.3)
	Female	17(68.0)	86(45.0)	103(47.7)
Address	Urban	11(44.0)	91(47.6)	102(47.2)
	Rural	14(56.0)	100(52.4)	114(52.8)
Occupation	Farmer	9(36.0)	49(25.7)	58(26.9)
	Merchant	3(12.0)	18(9.4)	21(9.7)
	Employed	3(12.0)	25(13.1)	28(13.0)
	Unemployed	8(32.0)	42(22.0)	50(23.1)
	Others	2(8.0)	57(29.8)	59(27.3)
Educational status	Literate	21(84.0)	174(91.1)	195(90.3)
	Illiterate	4(16.0)	17(8.9)	21(9.7)
Educational level	1-4 Grade	1(5.0)	21(12.1)	22(11.3)
	5-8 Grade	5(25.0)	48(27.6)	53(27.3)
	9-12 Grade	11(55.0)	71(40.8)	82(42.3)
	Above 12	3(15.0)	34(19.5)	37(19.1)
Ethnicity	Hadiya	18(72.0)	113(59.2)	131(60.6)
	Kambata	2(8.0)	27(14.1)	29(13.4)
	Silte	2(8.0)	23(12.0)	25(11.6)
	Gurage	2(8.0)	16(8.4)	18(8.3%)
	Others	1(4.0)	12(6.3)	13(6.0)
Religion	Protestant	8(32.0)	86(45.0)	94(43.5)
	Orthodox	5(20.0)	53(27.7)	58(26.9)
	Muslim	6(24.0)	39(20.4)	45(20.8)
	Catholic	3(12.0)	10(5.2)	13(6.0)
	Others	3(12.0)	3(1.6)	6(2.8)
Marital status	Married	11(44.0)	88(46.1)	99(45.8)
	Single	9(36.0)	85(44.5)	94(43.5)
	Widowed	3(12.0)	10(5.2)	13(6.0)
	Divorced	2(8.0)	8(4.2)	10(4.6)

### Behavioral and clinical characteristics of the study participants

Patients with schizophrenia accounted for 110 (50.9%) of the participants, followed by those with bipolar disorder (38 (17.6%) and major depressive disorder (41.0%). Just 14 (6.5%) of the total study participants had a family history of diabetes mellitus. Approximately 34 patients (15.7%) smoked, 28 patients (14.0%) chewed khat, 29 patients (13.4%) drank alcohol, and 114 patients (52.8%) engaged in light physical activity. Atypical antipsychotics 102(47.2%) were used to treat most of the individuals, followed by antipsychotics combined with antidepressants 43(19.9%). For mental health patients, the mean ± standard deviation of length of time they received antipsychotic therapy was 4.92± (3.969) years.(**Table 2).**

**Table 2:** Clinical and Behavioural Characteristics of psychiatric patients receiving antipsychotic treatment at the Wachamo University Nigist Elleni Mohammed Memorial Comprehensive Specialized Hospital, Hossana, SNNPR, Ethiopia, June 01 to August 30, 2022.

Variable	Category	Diabetes mellitus		Total No (%)
		Yes No (%)	No No (%)	
Family history of diabetes mellitus	Yes	2(8.0)	12(6.3)	14(6.5%)
	No	23(92.0)	179(93.7)	202(93.5)
Physical activity	Light	20(80.0)	94(49.2)	114(52.8)
	Moderate	5(20.0)	97(50.8)	102(47.2)
History of alcohol intake	Yes	5(20.0)	30(15.7)	35(16.2)
	No	20(80.0)	161(84.3)	181(83.8)
Current alcohol drink	Yes	5(20.0)	24(12.6)	29(13.4)
	No	20(80.0)	167(87.4)	187(86.6)
Frequency of alcohol drinking	Daily	1(20.0)	6(25.0)	7(24.1)
	Frequently	2(40.0)	13(54.2)	15(51.7)
	Rarely	2(40.0)	5(20.8)	7(24.1)
History of smoking	Yes	3(12.0)	31(16.2)	34(15.7)
	No	22(88.0)	160(83.8)	182(84.3)
Current smoking habit	Yes	3(12.0)	19(9.9)	22(10.2)
	No	22(88.0)	172(90.1)	194(89.8)
Frequency of smoking	Daily	1(33.3)	14(73.7)	15(68.2)
	Frequently	1(33.3)	4(21.1)	5(22.7)
	Rarely	1(33.3)	1(5.3)	2(9.1)
Family history of smoking	Yes	1(4.0)	18(9.4)	19(8.8)
	No	24(96.0)	173(90.6)	197(91.2)
Chat chewing habit	Yes	5(20.0)	23(12.0)	28(13.0)
	No	20(80.0)	168(88.0)	188(87.0)
Frequency of chat chewing	Daily	1(20.0)	4(17.4)	5(17.9)
	Most weekdays	3(60.0)	14(60.9)	17(60.7)
	Weekends only	1(20.0)	5(21.7)	6(21.4)
Types of psychiatry disorder	Schizophrenia	16(64.0)	94(49.2)	110(50.9)
	Schizoaffective disorder	1(4.0)	26(13.6)	27(12.5)
	MDD with Psychotic	3(12.0)	38(19.9)	41(19.0)
	Bipolar with Psychotic	5(20.0)	33(17.3)	38(17.6)
Type of antipsychotics	Typical	4(16.0)	29(15.2)	33(15.3)
	Atypical	13(52.0)	89(46.6)	102(47.2)
	Antipsychotic with Antidepressant	3(12.0)	40(20.9)	43(19.9)
	Antipsychotic with a mood stabilizer	5(20.0)	33(17.3)	38(17.6)
Duration of psychiatry disorder	≤3	3(12.0)	72(37.7)	75(34.7)
	4-6	8(32.0)	69(36.1)	77(35.6)
	≥7	14(56.0)	50(26.2)	64(29.6)
Duration of antipsychotic use	≤3	5(20.0)	93(48.7)	98(45.4)
	4-6	8(32.0)	61(31.9)	69(31.9)
	≥7	12(48.0)	37(19.4)	49(22.7)

### The prevalence of diabetes mellitus

The mean ± standard deviation of fasting blood glucose level was found to be 98.8 ± (22.71). The 2022 ADA criteria indicated that there were 25 cases (11.6%) of Type-2 diabetes mellitus. The prevalence of diabetes mellitus was higher in female participants (17(68.0%) vs. 8(32.0%)). The amount of DM rose in each of the five age groups: among individuals under 20, between 21 and 30, between 31 and 40, between 41 and 50, and above 50 years, respectively, 1(4.0%), 5(20.0%), 5(20.0%), 8(32.0%), and 6(24.0%) (**Table 1**). When it came to the psychiatric diseases that individuals with diabetes mellitus had, patients with schizophrenia had the highest prevalence of diabetes mellitus (16.0%), followed by patients with bipolar disorder, MDD with psychotic disorder, and schizoaffective disorder (5.0%, 3.0%), and 4.0%, respectively. Atypical medications such as Risperidone, Clozapine, and Olanzapine, 13(52.0%), were used to treat the majority of people with diabetes mellitus, followed by antipsychotics with antidepressants (Citalopram and Paroxetine), 5 (20.0%), and typical (Chlorpromazine and Haloperidol), 14 (16.0%) (**Table 2**). Of participants with diabetes mellitus, the prevalence of diabetes mellitus was higher in obese participants, 12(48.0), followed by overweight 6(24.0), and 19(76.0) participants were dyslipidemic (**Table 3**).

**Table 3:** Anthropometric measurements of psychiatric patients receiving antipsychotic treatment at the Wachamo University Nigist Elleni Mohammed Memorial Comprehensive Specialized Hospital, Hossana, SNNPR, Ethiopia, June 01 to August 30, 2022.

Variable	Category	Diabetes mellitus		Total No (%)
		Yes No (%)	No No (%)	
Waist circumstance for male	Normal	3(37.5)	88(69.3)	91(67.4)
	High	5(62.5)	39(30.7)	44(32.6)
Waist circumstance for female	Normal	7(41.2)	51(79.7)	58(71.6)
	High	10(58.8)	13(20.3)	23(28.4)
Waist circumstance for male and female	Normal	10(40.0)	139(72.8)	149(69.0)
	High	15(60.0)	52(27.2)	67(31.0)
Waist to Hip circumstance ratio for male	Normal	2(25.0)	64(50.4)	66(48.9)
	High	6(75.0)	63(49.6)	69(51.1)
Waist to Hip ratio circumstance for female	Normal	3(17.6)	42(65.6)	45(55.6)
	High	14(82.4)	22(34.4)	36(44.4)
Waist to Hip ratio for male and female	Normal	5(20.0)	106(55.5)	111(51.4)
	High	20(80.0)	85(44.5)	105(48.6)
Body mass index	Underweight	2(8.0)	12(6.3)	14(6.5)
	Normal	5(20.0)	148(77.5)	153(70.8)
	Overweight	6(24.0)	22(11.5)	28(13.0)
	Obesity	12(48.0)	9(4.7)	21(9.7)
Systolic pressure	Normal	15(60.0)	169(88.5)	184(85.2)
	High	10(40.0)	22(11.5)	32(14.8)
Diastolic blood pressure	Normal	17(68.0)	173(90.6)	190(88.0)
	High	8(32.0)	18(9.4)	26(12.0)
Hypertension	No	14(56.0)	164(85.9)	178(82.4)
	Yes	11(44.0)	27(14.1)	38(17.6)
Total Cholesterol	Normal	6(24.0)	167(87.4)	173(80.1)
	High	19(76.0)	24(12.6)	43(19.9)
HDL Cholesterol	Normal	13(52.0)	163(85.3)	176(81.5)
	Low	12(48.0)	28(14.7)	40(18.5)
LDL Cholesterol	Normal	8(32.0)	166(86.9)	174(80.6)
	High	17(68.0)	25(13.1)	42(19.4)
Triglyceride	Normal	12(48.0)	171(89.5)	183(84.7)
	High	13(52.0)	20(10.5)	33(15.3)
Dyslipidemia	No	6(24.0)	149(78.0)	155(71.8)
	Yes	19(76.0)	42(22.0)	61(28.2)

### Factors associated with diagnosed diabetes mellitus

In the bivariate analysis of variables with diabetes mellitus, sex, physical activity, age, duration of psychiatric disorder, duration of antipsychotic treatment use, total blood cholesterol, high-density lipoprotein, low-density lipoprotein, systolic blood pressure, diastolic blood pressure, body mass index, waist circumstance, waist to hip ratio and triglyceride were found to have a statistically significant association with the prevalence of diabetes mellitus, they are candidate variables for multivariate logistic regression with p-value <0.25 (**Table 4**).

**Table 4:** Bivariate analysis of factors associated with diabetes mellitus among psychiatric patients receiving antipsychotic treatment at the Wachamo University Nigist Elleni Mohammed Memorial Comprehensive Specialized Hospital, Hossana, SNNPR, Ethiopia, June 01 to August 30, 2022.

Variable	Category	COR (95% C.I.)	P-VALUE
Sex of participant	Male	1	
	Female	.385(.159−.936)	.035*
Age	–	–	.007*
	≤20	1	
	21-30	.943(.102-8.708)	.959
	31-40	1.000(.108-9.229)	1.000
	41-50	.277(.032-2.430)	.247
	≥50	.119(.012-1.149)	.066
Address	Urban	1	
	Rural	.863(.373-1.998)	.732
Occupational status	–	–	.294
	Farmer	1	
	Merchant	. 1.102(268-4.532)	.893
	Employed	1.531(.380-6.161)	.549
	Unemployed	.964(.342-2.722)	.945
	Others	5.235(1.079-25.390)	.040
Educational status	Literate	1	
	Illiterate	.513(.158-1.669)	.267
Educational level	–	–	.618
	1-4 grade	1	
	5-8 grade	.457(.050-4.156)	.487
	9-12 grade	.307(.037-2.521)	.272
	Abobe 12	.540(.053-5.534)	.604
Marital status		–	.455
	Married	1	
	Single	1.181(.466-2.992)	.726
	Widowed	.417(.099-1.749)	.232
	Divorced	.500(.094-2.660)	.416
Family history of DM	Yes	1	
	No	1.297(.273-6.164)	.744
Physical activity	Light	.242(.087−.672)	.006*
	Moderate	1	
History of alcohol intake	Yes	1	
	No	1.342(.467-3.852)	.585
Current alcohol drink	Yes	1	
	No	1.740(.597-5.068)	.310
Frequency of alcohol drink	–	–	.671
	Daily	1	
	Frequently	1.083(.081-14.412)	.952
	Rarely	.417(.029-6.064)	.522
History of smoking	Yes		
	No	.704(.198-2.496)	.587
Current smoking habit	Yes		
	No	1.234(.338-4.511)	.750
Frequency of smoking	–	–	.313
	Daily	1	
	Frequently	.286(.014-5.660)	.411
	Rarely	.071(.002-2.216)	.132
Family history of smoking	Yes	1	
	No	.400(.051-3.137)	.384
Khat chewing habit	Yes	1	
	No	1.826(.625-5.337)	.271
Frequency of khat chewing	–	–	.989
	Daily		
	Most week days	1.167(.094-14.518)	.905
	Weekends only	1.250(.058-26.869)	.887
Type of psychiatric disorder	–	–	.381
	Schizophrenia	1	
	Schizoaffective disorder	4.426(.560-34.947)	.158
	MDD with psychotics	2.156(.594-7.828)	.243
	Bipolar with psychotics	1.123(.382-3.307)	.833
**Type of antipsychotics**	**–**	–	.779
	Typical	1	
	Atypical	1.098(.269-4.482)	.896
	Antipsychotics with Antidepressant	1.037(.343-3.135)	.948
	Antipsychotics with a mood stabilizer	2.020(.449-9.088)	.359
Psychiatric disorder duration	–	–	.009*
	≤3	1	
	4-6	.359(.092-1.411)	.142
	≥7	.149(.041−.545)	.004*
Duration of antipsychotic use	–	–	.005*
	≤3	1	
	4-6	.410(.128-1.312)	.133
	≥7	.166(.055−.503)	.002
Waist circumstance for male and female	Normal	1	
	High	.249(.105−.590)	.002*
Waist to Hip ratio for male and female	Normal	1	
	High	.200(.072−.556)	.002*
Body mass index			.000*
	Underweight	1	
	Normal	4.933(.864-28.167)	.073
	Overweight	.611(.106-3.510)	.581
	Obesity	.125(.022−.704)	.018
Systolic blood pressure	Normal	1	
	High	.195(.078−.488)	.000*
Diastolic blood pressure	Normal	1	
	High	.221(.084−.584)	.002*
Hypertension	No	1	
	Yes	.210(.086−.509)	.001*
Total Cholesterol	Normal	1	
	High	.045(.016−.125)	.000*
HDL- Cholesterol	Normal	1	
	Low	.186(.077−.449)	.000*
LDL- Cholesterol	Normal	1	
	High	.071(.028−.181)	.000*
Triglyceride	Normal	1	
	High	.108(.043−.269)	.000*
Dyslipidemia	No	1	
	Yes	.089(.033−.237)	.000*

Note: *: significant p-value in a bi-variable model, **1**: reference

However, in the multivariate logistic regression analysis of the study variables, there was a statistically significant association only with sex, duration of antipsychotic treatment, body mass index, and total cholesterol. Our study revealed that females were found to be 6.483 times more likely at risk of having DM than males (AOR: 6.483 (1.647–25.516), and as the duration of antipsychotic use increased by one year, the risk of having DM increased by 7.876 units (AOR: 7.876 (1.587–39.090). A light physical activity lifestyle was found to be 0.220 times at risk of having diabetes mellitus than a moderate physical activity lifestyle (AO: 0.220 (057-.852), and body mass index and total cholesterol increased by 1 kg/m^2^ and 1 mg/dl, the risk of having diabetes mellitus increased by 11.869 (2.188–64.374), and 13.742 (3.153–59.887) respectively(**Table 5).**

**Table 5:** Multivariate analysis of factors associated with diabetes mellitus among psychiatric patients receiving antipsychotic treatment at the Wachamo University Nigist Elleni Mohammed Memorial Comprehensive Specialized Hospital, Hossana, SNNPR, Ethiopia, June 01 to August 30.2022.

Variable	Category	COR(95% C.I)	ACOR(95% C.I)	Adgusted P –value
Sex	Male		1	
	Female	.385(.159−.936)	6.483(1.647, 25.516)	.007
Physical activity	Light	.242(.087.672)	1	
	Moderate		.220(.057−.852)	.028
Duration of antipsychotic use				.030
	≤3		1	
	4-6	.410(.128-1.312)	7.876(1.587-39.090)	.012
	≥7	.166(.055−.503)	1.258(.317-4.984)	.744
Body mass index				.008
	Underweight		1	
	Normal	4.93(.864-28.167)	1.067(.096-11.854)	.958
	Overweight	.611(.106-3.510)	11.869(2.188-64.374)	.004
	Obesity	.125(.022−.704)	1.527(.302-7.724)	.609
Total Cholesterol	Normal		1	
	High	.108(.043−.269)	13.74(3.153-59.887)	<0.001

## Discussion

In this study, of 216 psychiatric patients, 25(11.6%) met the WHO criteria for diabetes mellitus. The prevalence of type 2 diabetes mellitus (11.6%) found in this study was higher than the estimated diabetes mellitus evidence from the National non-communicable diseases(NCD) Survey, 3.2% reported by the NCDs in 2015 [27]. This may be associated with the diabetogenic effect of antipsychotic drugs and the global increase in the trend of the DM epidemic in developing countries. The prevalence of type 2 diabetes mellitus among patients with a psychiatric disorder in this study, (11.6%), was lower than the findings of the studies among psychiatric patients in china (20.5%) [28], Barcelona (16%) [29], Pakistan (20.3) [4] and Kano state of Nigeria(24.2%) [10]. The differences observed in these studies were likely having a higher national prevalence of diabetes mellitus which could be due to lifestyle and genetic factors. On the contrary, the prevalence (11.6%) of diabetes mellitus was also higher than the study finding in psychiatric patients in South Africa(3.9%) [30], Gondar, 7.3% [1], and Hawassa (6.3%)[9s]. This could be due to an absence in DM screening and early diagnosis because of insufficient resources and practical considerations. And also this study is consistent with the study conducted on psychiatric patients in the USA (10.2%) [31], Uganda(9.87) [32], and Kaduna, northern Nigeria (12.8%) [33].

A higher prevalence of diabetes mellitus was observed in females 17(68.0%) than in males 8(32.0%), which is consistent with a study carried out on psychiatric patients in China (35 vs 21.53 [28], Uganda (44.41% were male and 55.59% were female [32], Gondar (5.9%) vs (1.5%) [1]. The possible reason for a highly represented DM among female psychiatric patients in this study may be a lack of physical exercise.

Regarding psychiatric disorders, the prevalence of diabetes mellitus was higher in schizophrenic16 (64.0) patients treated by atypical antipsychotic treatment13 (52.0). The finding of this study is similar to the study conducted in Kano state of Nigeria (37.6%) [10], Gondar 106 (51.7%)[1s], and Hawassa 8 (3.4%) [9].

The results of my study revealed that gender, duration of antipsychotic treatment use, physical activity, and total cholesterol were significant associations with the prevalence of diabetes mellitus. Factors like age, duration of psychiatric disorder, systolic blood pressure, diastolic blood pressure, high-density lipoprotein, low-density lipoprotein, and triglyceride were not significantly associated with diabetes mellitus.

Diabetes mellitus was significantly associated with sex, physical activity, duration of antipsychotic treatment use, body mass index, and total cholesterol. Our study revealed that females were found to be 6.483 times more likely at risk of having DM than males (AOR: 6.483 (1.647–25.516). The finding of this study was also supported by a study done in Gondar, which shows females were found to be 7.1 times to be at risk of having DM than males (AOR: 7.1, CI: 1.4–36.1) [1].

The duration of antipsychotic use increased by one year, and the risk of having DM increased by 7.876 units (AOR: 7.876 (1.587–39.090). The finding of this study was also supported by a study done in Gondar, which showed an association between the duration of antipsychotic treatment and an increased risk of diabetes mellitus (AOR: 1.47, CI: 1.021–2.125) [1]. The diabetogenic effects of a longer duration of antipsychotic treatment were on antipsychotic-induced weight gain. This starts with the eventual development of insulin resistance and pancreatic beta-cell failure

A moderate physical activity lifestyle was found to make 0.220 times less likely at risk of having diabetes mellitus than a light physical activity lifestyle (AO: 0.220 (057-.852), and body mass index and total cholesterol increased by 1 kg/m^2^ and 1 mg/dl, the risk of having diabetes mellitus increased by 11.869 (2.188–64.374), and 13.742 (3.153–59.887) respectively. Similar studies reported the association of diabetes mellitus with physical activity, body mass index, and total cholesterol [4,10,15,28]. The alterations in the depicted parameters and physical inactivity in psychiatric patients might be suggestive that they develop diabetes complications, despite controlling the severity of mental problems through antipsychotic treatments.

### Limitations of the study

The general adult population was not used as the control group in this study, but we did attempt to compare our results with those of other studies on this population. In terms of diabetes, the study found a high prevalence of diabetes mellitus, higher than that reported in the general population. Because the study is cross-sectional, it is not able to adequately express the evidence of diabetes and its causal risk factors. We diagnosed diabetes with FBS; an oral glucose tolerance test was not performed because of the patient's psychosis and procedural challenges. Also sampling method was consecutive, it possible to be a selection bias. Regardless of the restrictions that are illustrated, this study ultimately contributes positive data to Ethiopia limited data situation.

## Conclusion

The findings of the present study revealed that the prevalence of diabetes mellitus was high among psychiatric patients taking antipsychotics. Sex, physical activity, body mass index, and total cholesterol were significantly associated with diabetes mellitus. Based on the results obtained from the study, the following recommendations were forwarded. It is recommended that investigators carry out carefully monitored cohort studies to evaluate the long-term impact of antipsychotic medication and the illness on blood glucose levels by determining HgbA_1_c. Healthcare professionals should test and diagnose diabetes mellitus in all mentally ill patients early, especially those who are older and have been using antipsychotic medications for a long period. It will be crucial to undertake lifestyle interventions to lower the risk of complications connected to diabetes mellitus, such as cardiovascular illnesses.

## Supporting information

S1 File Minimal data sets. (SAV)
